# Metagenomic next-generation sequencing for the diagnosis of *Pneumocystis jirovecii* Pneumonia in critically pediatric patients

**DOI:** 10.1186/s12941-023-00555-5

**Published:** 2023-01-16

**Authors:** Hengxin Chen, Yujian Liang, Ruizhi Wang, Yijie Wu, Xiaoyun Zhang, Hao Huang, Xuegao Yu, Mengzhi Hong, Juhua Yang, Kang Liao, Hongxu Xu, Min Liu, Peisong Chen, Yili Chen

**Affiliations:** 1 Department of Laboratory Medicine, The First Affiliated Hospital of Sun Yat-Sen University, Guangzhou, 510080 Guangdong China; 2 Department of Pediatric Intensive Care Unit, The First Affiliated Hospital of Sun Yat-Sen University, Guangzhou, 510080 Guangdong China; 3 Zhongshan School of Medicine, Sun Yat-Sen University, Guangdong, China; 4 Vision Medicals Co., Ltd, Guangzhou, China

**Keywords:** Metagenomics next generation sequencing, *Pneumocystis jirovecii* pneumonia, Critically pediatric patients, Diagnosis

## Abstract

**Objective:**

The aim of this study was to evaluate the effectiveness of metagenomic next-generation sequencing (mNGS) for the diagnosis of *Pneumocystis jirovecii* Pneumonia (PCP) in critically pediatric patients.

**Methods:**

Seventeen critically pediatric patients with PCP and sixty patients diagnosed with non-PCP pneumonia who were admitted in pediatric intensive care unit between June 2018 and July 2021 were enrolled. Conventional methods and mNGS for detecting *Pneumocystis jirovecii* (*P. jirovecii*) were compared. The patients’ demographics, comorbidities, laboratory test results, antibiotic treatment response and 30 day mortality were analyzed.

**Result:**

The mNGS showed a satisfying diagnostic performance with a sensitivity of 100% in detecting *P. jirovecii* compared with Gomori methenamine silver staining (5.9%), serum (1,3)-β-D-glucan (86.7%) and and LDH (55.6%). The diagnostic specificity of mNGS for PCP was higher than that of serum BDG (56.7%) and LDH (71.4%). In PCP group, over one thirds’ cases had mixed infections. Compared with survivors, non-survivors had higher stringently mapped read numbers (SMRNs) in bronchoalveolar lavage fluid (BALF) sample (*P* < 0.05), suggesting SMRNs were closely associated with the severity of response. The detection for *P. jirovecii* by mNGS both in BALF and blood samples reached a concordance rate of 100%, and the SMRNs in the BALF were remarkably higher than that in blood samples. Initial antimicrobial treatment was modified in 88.2% of PCP patients based on the mNGS results.

**Conclusion:**

The mNGS is a potential and efficient technology in diagnosing PCP and shows a satisfying performance in the detection of co-pathogens. Both blood and BALF samples for mNGS are suggested for the presumptive diagnosis of PCP.

**Supplementary Information:**

The online version contains supplementary material available at 10.1186/s12941-023-00555-5.

## Introduction

*Pneumocystis jirovecii* (*P. jirovecii*) is a common opportunistic infection which causes *Pneumocystis jirovecii* pneumonia (PCP) in immunocompromised population [1]. PCP is the important cause of death in hospitalized adults (13%) and children (29%) among HIV-infected people [2]. In recent years, there is an increasing incidence of PCP related to non-HIV patients, such as underlying malignancy, post-organ transplantation, kwashiorkor, treatment-related immunosuppression and/or concomitant use of corticosteroids [3, 4]. In children, the mortality of PCP with leukemia is 28–53%, and 17–30% in children with AIDS [5, 6]. Without chemoprophylaxis, up to 25% of pediatric oncology patients receiving chemotherapy will develop PCP [7]. PCP is one of the most common infectious diseases that cause children to die [8].

The diagnosis of PCP requires a combination of clinical manifestations, radiological findings and microbiological tests [9–12]. All signs and symptoms of PCP are non-specific. When the immunosuppressive host clinically presents chills, dry cough, shortness of breath, weight loss and progressive dyspnea, the possibility of PCP should be given priority [13]. Notably, compared to children older than 6 months, clinical progression of PCP in the children aged one to six months is slower [4]. The non-specific feature increases the complexity of PCP diagnosis in critically pediatric patients. Therefore, accurate and rapid diagnosis is essential for the prognosis of PCP patients.

Currently, the laboratory identifications of *P. jirovecii* contain classical morphology examinations and molecular methods [14]. *P. jirovecii* still cannot be reliably grown in vitro [15]. According to the characteristic cysts and trophozoites found under the specific staining of bronchoalveolar lavage fluid (BALF), induced sputum and other specimens, the microscopic examination of respiratory tract specimens can be used as the gold standard for the diagnosis of PCP. However, the sensitivity of routine staining is low, and its negative report is not enough to exclude the diagnosis of PCP [16]. Moreover, immunofluorescence staining is not routinely performed in many hospitals. Recently, the polymerase chain reaction (PCR) method has been considered a promising technology for detecting *P. jirovecii*, which has an excellent sensitivity (94–99%) and specificity (89–93%), even in specimens with low pathogen load [17]. However, due to the limitation of the genus-specific targeting regions primers, PCR methods still have difficulty in identifying mixed infections [18, 19].

Metagenomics next-generation sequencing (mNGS) is an unbiased pathogen detection and molecular technology of nucleic acid sequencing with high-throughput in a single assay, which has been considered as a promising microbial identification technology in infectious diseases [20]. Recently, its diagnostic ability on detecting a wide range of pathogens has been highlighted in several studies [21, 22]. We once reported a case about rapid and precise diagnosis of pneumonia coinfected by *P. jirovecii* and *Aspergillus fumigatus* assisted by next-generation sequencing in a patient with systemic lupus erythematosus [23]. However, the diagnostic performance of mNGS for PCP by BALF and blood specimens in non-HIV critically pediatric patients has rarely been reported.

In our study, we described the performance of mNGS of BALF and blood samples for detecting *P. jirovecii* in non-HIV critically pediatric patients.

Methods

### Study participants

**Fig. 1:**
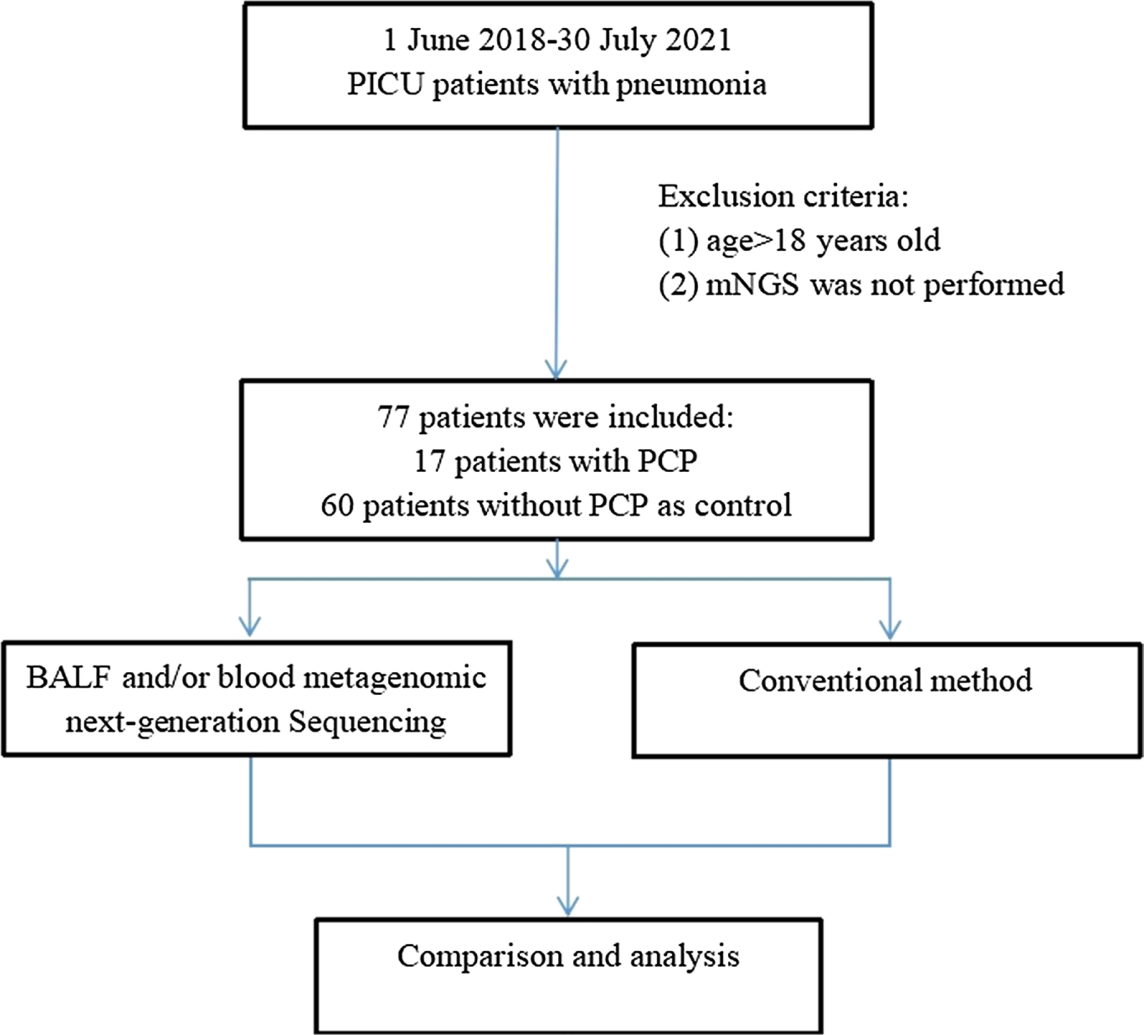
Flowchart of case selection. A total of 77 pneumonia cases in PICU were selected for further analysis. *PICU* pediatric intensive care unit

In this retrospective study, we consecutively enrolled 77 pneumonia patients who were admitted to the pediatric intensive care unit (PICU) of The First Affiliated Hospital of Sun Yat-sen University, from June 1st, 2018 to July 30th, 2021. According to the European Organization for Research and Treatment of Cancer and the Mycoses Study Group Education and Research Consortium (EORTC/MSGERC) consensus definitions of invasive fungal diseases (IFDs) [24], the patients were divided into an observation group (PCP proven and probable diagnosed group) and a control group (non-PCP group). The details were as follows: (1) non-HIV immunosuppressed hosts; (2) accompanied by fever or dry cough, shortness of breath; (3) Chest computed tomography (CT) showed multiple ground-glass interstitial exudation, reticulate or consolidated shadows in both lungs; (4) serum (1,3)-β-D-glucan (Serum BDG) positive (> 60 pg/ml) twice; and (5) *P. jirovecii* trophozoites (and/or cysts) were microscopically identified following Gomori methenamine silver staining. Clinical diagnosis was made if the aforementioned items (1)–(4) were met, and confirmed diagnosis was made if items (1)–(5) were met. The clinical comprehensive diagnosis of PCP or non PCP was made by two senior expert pulmonary doctors (YJL and WT) after discussion with the medical team based on clinical symptoms, laboratory findings, chest radiology, microbiological tests and treatment responses. Patients were excluded if they met any of the following criteria: (1) HIV infection; (2) mNGS was not performed; (3) age > 18 years old; (4) medical record was incomplete (Fig. 1). The study was approved by The First Affiliated Hospital of Sun Yat-sen University and was in line with the Declaration of Helsinki.

### Sample collection and etiological diagnosis

BALF was collected according to the guidelines [25]. After elimination of contraindications, all patients underwent bronchoscopy under intravenous combined anesthesia or 2% lidocaine topical anesthesia. After the same amount of normal saline was injected into the affected bronchial segment in several times, BALF was aspirated under negative pressure for relevant tests.

At the same time, BALF mNGS and conventional methods were used to detect pathogens in all patients. BALF and peripheral blood specimens were simultaneously submitted for etiological examination. In this study, microbiologic tests for *P. jirovecii* included serum (1,3)-β-D-glucan (Serum BDG), lactate dehydrogenase (LDH), Gomori methenamine silver (GMS) staining and mNGS. Other etiological laboratory diagnosis also included traditional culture methods and antigen or antibody detections.

### Sample processing and DNA extraction for mNGS

Volumes of 600 µL of each patient’s BALF was taken and mixed with lysozyme and glass beads. Then, the mixture was placed inside a vortex mixer's horizontal platform and stirred intensely for 30 min at 2800–3200 rpm. For nucleic acid extraction from BALF, we transferred 200 µL of supernatant into a 2 mL centrifuge tube. Volumes of 3–5 mL of patients’ blood were centrifuged at 3500 rpm for 10 min at 4 °C for plasma separation. For DNA extraction from blood, we transferred 200 µL of plasma into a 2 mL sterile tube. Then the IDseq TM Micro DNA kit (Vision medicals, VM002-50D, China) was used to extract DNA based on standard procedures [26].

### Library preparation and sequencing construction

DNA libraries were builded via transposase-based methodology. After purification and size selection, the concentration of the library was measured by using a Qubit instrument before pooling. Pooled libraries were sequenced on an Illumina NextSeq 550 system using a 75 bp, single-end sequencing kit. The qualified results had no fewer than 10 million reads obtained per sample and a Q30 score of 85% or greater. A negative control sample was processed and sequenced in parallel in each sequencing run for quality control [27].

### Bioinformatic analysis

High-quality sequencing data were generated by removing low-quality and short (length < 35 bp) reads using fastp software [28]. Human host sequences were subtracted by mapping to human reference genome sequences (National Center for Biotechnology Information GRCh38 assembly) using the Burrows-Wheeler Aligner tool (BWA) [29]. After the removal of low-complexity reads, the remaining data were classified by alignment to curated microbial genome databases for viruses, bacteria, fungi, and parasites. We developed a set of criteria similar to the National Center for Biotechnology Information (NCBI) (https://www.ncbi.nlm.nih.gov/genome/) criteria for selecting the representative assembly for microorganisms. After each microorganism is quantified, it is important to remove the contamination from the reagents. To determine the list of background microorganisms, we classified microorganisms detected in at least 25% of the samples, including negative controls. The quantitative value of the abundance of each microorganism and the total amount of nucleic acid were tested for correlation. When the quantitative value of the abundance of this microorganism was negatively correlated with the total amount of nucleic acid in the sample, it was determined as the reagent-derived background organisms.Therefore, they were excluded from the report. The sequencing data list was analyzed in terms of stringently mapped read number (SMRN, representing a species specific sequence), genome coverage (%) and relative abundance (%).

### Threshold criteria for interpretation of metagenomic analysis

The microbial list obtained from the above analysis process was compared with an in-house background database, which contains microorganisms appearing in more than 50% samples in the laboratory in the past three months. The suspected background microorganisms were removed from the microbial list. For different types of microbes, the thresholds were set as follows: (1) Extracellular bacteria/fungus (excluding *Cryptococcus*)/parasites: SMRN ≥ 30, ranked among the top 10 for bacteria, fungi, or parasites. Organisms detected in the negative control group or that were present in ≥ 25% of samples from the previous 30 days were excluded but only if the detected SMRN was ≥ tenfold than that in the negative control group or other organisms. In addition, organisms present in ≥ 75% of samples from the previous 30 days were excluded. (2) Intracellular bacteria (excluding *Mycobacterium tuberculosis* and *Brucella*)/ *Cryptococcus*: SMRN ≥ 10, ranked among the top 10 for bacteria or fungi. Pathogens detected in the negative control group or that were present in≥ 25% of samples from the previous 30 days were excluded but only if the detected SMRN was ≥ tenfold than that in the negative control group or other organisms. (3) Virus/*Brucella*: SMRN ≥ 3, Pathogens detected in the negative control group were excluded but only if the detected SMRN was ≥ tenfold than that in the negative control group. (4) *Mycobacterium tuberculosis*: SMRN ≥ 1 [26, 30].

### qPCR assay of *P. jirovecii* for validation experiment

The sequence of the primer pairs used is listed as follows: PjF (5′ -GCACGTTGGCCTCGTTTAC-3′) and PjR (5′ -GATGAAGCTCACTTTCCGATGAC-3′). The primers used in this qPCR assay target a 157 bp fragment. The qPCR assay was performed on Applied Biosystems 7500 Fast PCR system. The final reaction volume of 25 µL contained 12.5 µL of TaqMan Universal Master Mix (InvitrogenTM), MgCl2 (1.5 mL), 0.4 mM concentration of each primer (1 mL), 0.2 mM probe (0.5 mL) and 8.5 µL of extracted DNA. Thermal cycling conditions were as follows: preheating at 95 °C for 10 min, amplification of 45 cycles including denaturation at 95 °C for 20 s, annealing and extension at 60 °C for 1 min. Positive, negative, and extraction controls were included in each run. The CT value for positive samples was recorded for each run.

### Clinical data collection

Clinical parameters of each patient were acquired through review of electronic medical records. We recorded patient data regarding demographics, pediatric risk of mortality score (PRISM), pediatric critical illness score (PCIS), underlying diseases, the length of ICU stay, use of immunosuppressant, laboratory test results, antibiotic treatment response and 30 days mortality.

### Statistical analysis

Statistical analysis was carried out by an online statistics tool (http://dxonline.deepwise.com/) and Graphpad prism. Continuous variables were presented as medians and interquartile ranges and categorical variables as counts and percentages. The Wilcoxon Test was used for comparing the differences of continuous variables between PCP and non-PCP group and the X^2^ test for categorical variables. Spearman correlation test was used for analyzing correlation between the stringently mapped read numbers (SMRNs) of *P. jirovecii* detected by mNGS and LDH, serum BDG levels, pediatric risk of mortality (PRISM) as well as pediatric critical illness score (PCIS). Sensitivity, specificity, likelihood ratio, Jouden index, positive predict value (PPV) and negative predict value (NPV) was calculated using the clinical composite diagnosis as the reference standard. Significance was fixed at *P* < 0.05.

## Results

### Clinical characteristics and laboratory findings

There were 77 patients with severe pneumonia in this study, including 17 patients with PCP and 60 without PCP. The median age (4.3 years vs. 3 years), sex composition, pediatric risk of mortality (PRISM) and pediatric critical illness score (PCIS) were similar between the PCP group and the non PCP group. Patients with PCP mainly had dyspnea (88.2%), fever (76.5%) and cough (41.2%). Obviously, the PCP group was more prone to dyspnea than the non-PCP group. Various immunosuppressive conditions occurred in both groups. Corticosteroid use (41.2%), hematological malignancies (41.3%), and solid tumors (41.4%) all ranked first in PCP patients.

Compared with the non-PCP group, ARDS, mechanical ventilation and indwelling urinary catheter were more common in PCP. Days of mechanical ventilation in PCP group were longer. Peripheral blood lymphocyte, a median count of 0.34X10^9^/L, was significantly lower in PCP patients. Serum BDG and LDH were significantly higher in PCP patients, respectively (*P* < 0.05). Ground-glass opacity was significantly more frequent in PCP patients (*P* < 0.05). In 33 ARDS patients, 14 patients were diagnosed with PCP by mNGS (Table 1).

**Table 1:** The basic clinical data of enrolled patients

Characteristics (median[IQR] or n[%]	PCP patients (n = 17)	Non-PCP patients (n = 60)	*P* value
Age (years)	4.50 (3.00–7.00)	3.00 (1.00–8.00)	0.164
Male	11.00 (64.70%)	37.00 (61.70%)	0.891
PRISM	12.50 (10.00–15.00)	13.00 (10.00–17.50)	0.992
PCIS	82.00 (79.50–90.00)	87.00 (84.00–93.00)	0.100
Clinical symptoms			
Fever	13.00 (76.50%)	42.00 (71.20%)	0.903
Cough	7.00 (41.20%)	17.00 (28.80%)	0.334
Dyspnea	15.00 (88.20%)	16.00 (27.10%)	0.000*
Immunocompromised conditions			
Use of corticosteroids	7.00 (41.20%)	25.00 (42.40%)	0.930
Use of immunosuppressive agents	1.00 (5.90%)	2.00 (3.40%)	1.000
Hematologic malignancies	7.00 (41.20%)	23.00 (38.30%)	0.832
Solid tumors	7.00 (41.20%)	19.00 (31.70%)	0.464
Rheumatic diseases	2.00 (11.80%)	0.00 (0.00%)	0.067
Chest CT images			
Ground-glass opacity	10.00 (58.80%)	1.00 (1.90%)	0.000*
Patchy shadowing	11.00 (64.70%)	36.00 (66.70%)	0.882
Interstitial patterns	3.00 (17.60%)	25.00 (46.30%)	0.035*
Pleural effusion	1.00 (5.90%)	13.00 (24.10%)	0.195
Cystic changes	1.00 (5.90%)	0.00 (0.00%)	0.539
Indwelling gastric tube	15.00 (88.20%)	45.00 (76.30%)	0.466
Indwelling urinary catheter	14.00 (82.40%)	33.0 (55.90%)	0.048*
Indwelling vein catheter	15.00 (88.20%)	48.00 (81.40%)	0.766
Mechanical ventilation	16.00 (94.10%)	34.00 (57.60%)	0.005*
Days of mechanical ventilation	7.00 (5.00–8.50)	4.00 (0.00–9.00)	0.038*
Serum BDG (ng/L)	435.16 (152.21–600.00)	48.04 (37.50–153.90)	0.027*
LDH (U/L)	799.00 (566.00–921.00)	354.50 (236.25–712.50)	0.002*
CRP (mg/L)	57.32 (15.91–90.75)	41.13 (10.86–125.735)	0.601
PCT (ng/mL)	0.37 (0.22–0.52)	0.71 (0.27–7.06)	0.085
White blood cells (* 10^9^/L)	4.20 (2.07–10.91)	6.39 (0.615–11.78)	0.971
Neutrophils (*10^9^/L)	3.18 (1.15–8.66)	2.59 (0.115–7.598)	0.576
Lymphocytes (*10^9^/L)	0.34 (0.1–0.86)	1.05 (0.36–3.65)	0.025*
CRRT	2.00 (11.80%)	5.00 (8.30%)	1.000
ARDS	14.00 (82.40%)	19.00 (31.70%)	0.000*
LOS in hospital	13.00 (9.00–16.00)	18.50 (9.75–40.25)	0.060
LOS in PICU	10.00 (8.00–14.00)	11.50 (7.75–20.00)	0.337
30 days-mortality	2.00 (11.80%)	10.00 (16.90%)	0.889

*IQR* interquartile range, *PRISM* pediatric risk of mortality score, *PCIS* pediatric critical illness scoring, Serum BDG Serum (1,3)-β-D-glucan, *CT* computed tomography, *LDH* lactate dehydrogenase, *CRP* C-reactive protein, *PCT* procalcitonin, *CRRT* continuous renal replacement therapy, *ARDS* acute respiratory distress syndrome, *LOS* length of stay

^*^P < 0.05

### Performance comparison between mNGS and other diagnostic methods

The *Ct* value of *P. jirovecii* qPCR and mNGS sequencing results of 17 PCP cases in this study were listed in Table 2. Our result showed that the *Ct* value of all the cases with PCP detected by mNGS was less than 40, suggesting that the detection of PCP by mNGS was reliable.

**Table 2:** The Ct value of *P. jirovecii* qPCR and mNGS sequencing results of 17 cases in this study

Case No.	Sample type	Specific reads of mNGS results (n)	*Ct* value
1	Blood	*Pneumocystis jirovecii* (712)	33.63
2	Blood	*Pneumocystis jirovecii* (27), *Parvovirus B19* (121)	32.54
	BALF	*Pneumocystis jirovecii* (284), *Acinetobacter baumannii* (527)	32.54
3	BALF	*Pneumocystis jirovecii* (7854)	31.13
4	BALF	*Pneumocystis jirovecii* (1104)	24.48
5	BALF	*Pneumocystis jirovecii* (367)	31.13
6	BALF	*Pneumocystis jirovecii* (5029)	34.03
7	BALF	*Pneumocystis jirovecii* (109,593), *CMV* (42), *Aspergillus fumigatus* (833), *EB* (18)	27.75
8	BALF	*Pneumocystis jirovecii* (487)	35.39
9	BALF	*Pneumocystis jirovecii* (9550), *TTV* (60), *Staphylococcus epidermidis* (18)	31.80
10	BALF	*Pneumocystis jirovecii* (1435)	32.71
11	Blood	*Pneumocystis jirovecii* (79), *Streptococcus pneumoniae* (27), *Escherichia coli* (53)	32.15
12	Blood	*Pneumocystis jirovecii* (6), *Pseudomonas aeruginosa* (83), *Acinetobacter baumannii* (359)	35.25
13	BALF	*Pneumocystis jirovecii* (871), *parvovirus B19* (62)	33.12
	Blood	*Pneumocystis jirovecii* (187), *Parvovirus B19* (161,506)	35.87
14	BALF	*Pneumocystis jirovecii* (22,576), *Streptococcus pneumoniae* (168)	30.12
15	BALF	*Pneumocystis jirovecii* (27,969)	28.00
16	Blood	*Pneumocystis jirovecii* (3024)	34.78
17	Blood	*Pneumocystis jirovecii* (1594), *Corynebacterium matruchotii* (106), *CMV* (72)	32.00

The diagnostic performance of serum BDG and mNGS were compared in 17 PCP patients in our study. As illustrated in Table 3, blood and/or BALF from all patients were conducted by mNGS. The sensitivity and specificity of mNGS was 100% and 96.7%, which was remarkably higher than serum BDG (86.7% and 56.7%) and LDH (55.6% and 71.4%). The sensitivity of mNGS was higher than GMS staining (5.8%). We also found that PPV (89.5%) and NPV (100%) of the mNGS overtook that of serum BDG and LDH.

**Table 3:** Diagnostic performance of mNGS, GMS staining, serum LDH and BDG for PCP

Methods	PC group	Non-PCP group	Sensitivity	Specificity	PPV	NPV
mNGS						
+	17	2	100.0%	96.7%	89.5%	100.0%
−	0	58	(77.1–100.0)	(87.5–99.4)	(65.5–98.2)	(92.3–100.0)
Serum BDG						
+	13	13	86.7%	56.7%	50.0%	89.5%
−	2	17	(58.4–97.7)	(37.7–74.0)	(30.4–69.6)	(65.5–98.2)
GMS						
+	1	0	5.9%	100.0%	100.0%	11.1%
−	16	2	(0.3–30.8)	(19.8–100)	(5.5–100)	(1.9–36.1)
LDH [30]						
≥ 618 U/L	10	16	55.6%	71.4%	38.5%	83.3%
< 618 U/L	8	40	(31.4–77.6)	(57.6–82.3)	(20.9–59.3)	(69.2–92.0)

*GMS* staining gomori methenamine silver staining, serum BDG serum (1,3)-β-D-glucan, *LDH* lactate dehydrogenase, serum BDG < 80 ng/L was defifined as positive, *LDH* < 618 U/Lwas defifined as positive, *CI* confifidence intervals, *PPV* positive predict value, *NPV* negative predict value

### mNGS for detection of *P. jirovecii* in blood and/or BALF samples

**Fig. 2:**
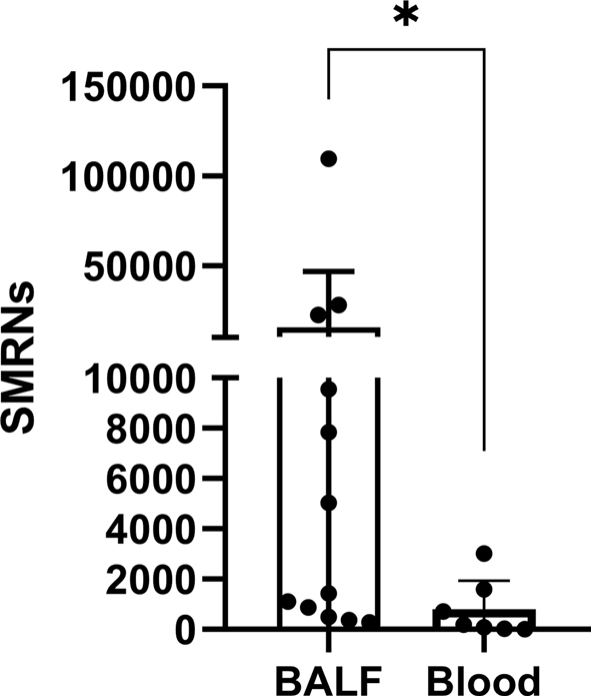
Comparison of the SMRNs of P. *jirovecii* detected by mNGS between BALF samples and blood samples

**Fig. 3:**
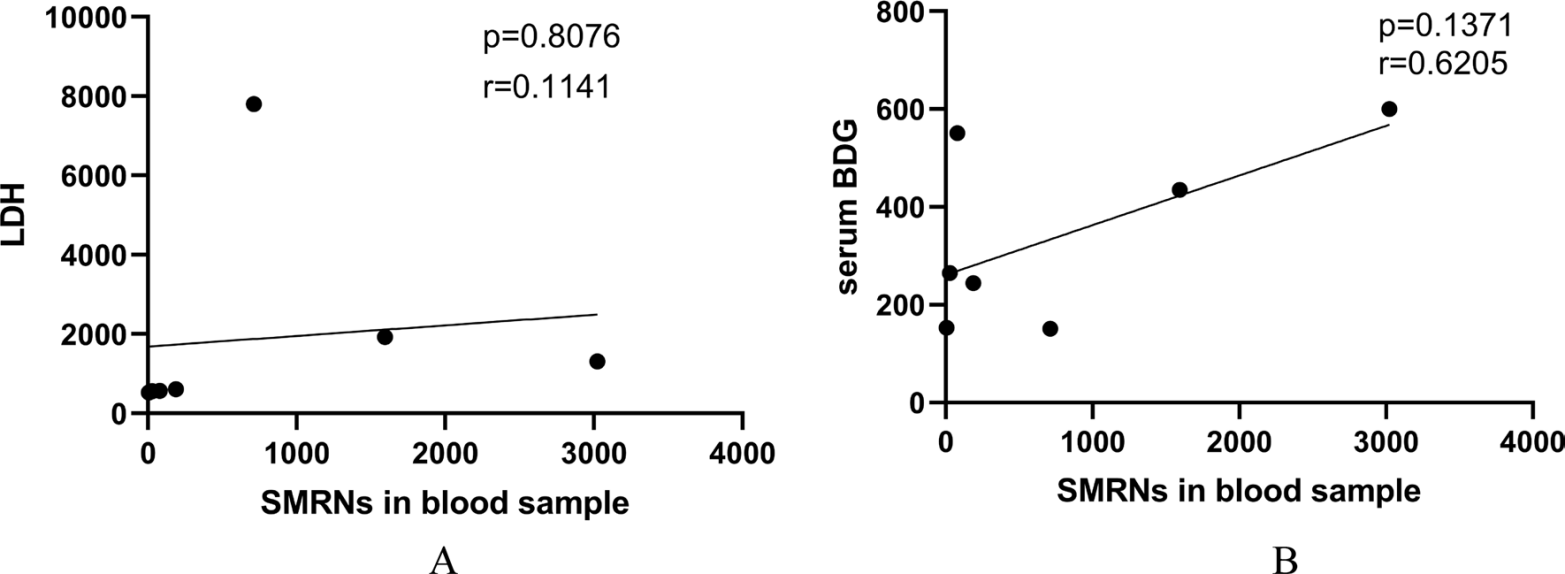
Correlation between the SMRNs of *P. jirovecii* detected by mNGS and serum LDH levels as well as serum BDG level

In total, there were 12 BALF samples and 7 blood samples in the patients with PCP (Table 4). Compared with survivors, non-survivors had higher read of SMRNs in BALF (*P* < 0.05). It is worth noting that the SMRNs in the BALF were significantly higher than that in blood samples (Fig. 2, 3232.00vs. 187.00, *P* = 0.022). Besides, the SMRNs of *P. jirovecii* detected by mNGS has a positive trend with serum BDG in blood (Fig. 3, R = 0.62, *P* > 0.05). Furthermore, we found non-survivors' lengths of stay in PICU were longer, but PRISM and PCIS in non-survivors were similar with survivors (Table 5). There was no correlation between the SMRNs of *P. jirovecii* and PRISM, PCIS as well as LDH.

**Table 4:** mNGS for detection of *P. jirovecii* in blood and/or BALF Samples from PCP group

Specimen	Patient nubmer	Positive	Negative
BALF only	10	10	0
Blood only	5	5	0
Both	2	2	0

**Table 5:** The wilcoxon test and the chi-square test analysis of risk factors for 30-days mortality

Characteristics (median[IQR] or n[%]	Non-survivors (n = 2)	Survivors (n = 15)	*P* value
Age (years)	9.50 (7.25–11.75)	4.00 (3.00–7.00)	0.204
Male	0.00 (0.00%)	11.00 (73.30%)	0.110
PRISM	13.50 (12.75–14.25)	12.50 (10.00–15.00)	0.808
PCIS	83.00 (78.50–87.50)	82.00 (80.00–89.50)	1.000
Immunocompromised conditions			
Use of corticosteroids	1.00 (50.00%)	6.00 (40.00%)	1.000
Use of immunosuppressive agents	1.00 (50.00%)	0.00 (0.00%)	0.118
Hematologic malignancies	1.00 (50.00%)	6.00 (40.00%)	1.000
Solid tumors	1.00 (50.00%)	6.00 (40.00%)	1.000
Rheumatic diseases	0.00 (0.00%)	2.00 (13.30%)	1.000
Indwelling gastric tube	1.00 (50.00%)	14.00 (93.30%)	0.228
Indwelling urinary catheter	1.00 (50.00%)	13.00 (86.70%)	0.331
Indwelling vein catheter	2.00 (100.00%)	13.00 (86.70%)	1.000
LDH (U/L)	905.50 (901.75–909.25)	630.00 (547.00–968.50)	0.618
CRP (mg/L)	119.11 (67.51–170.70)	57.32 (21.04–82.19)	0.824
PCT (ng/ml)	1.05 (0.58–1.53)	0.37 (0.23–0.49)	1.000
White blood cells (X 10^9^/L)	8.13 (6.07–10.18)	4.20 (1.84–10.64)	0.529
Neutrophils (X 10^9^/L)	6.04 (3.45–8.64)	3.18 (1.25–7.46)	0.824
Lymphocytes (X 10^9^/L)	0.56 (0.42–0.70)	0.34 (0.09–1.17)	0.824
SMRNs in BALF	68,781.00 (48,375.00–89,187.00)	1269.50 (583.00–7147.75)	0.030*
ARDS	14.0 (82.4%)	12 (80.0%)	1.000
Mechanical ventilation	2.00 (100.00%)	14.00 (93.30%)	1.000
Days of mechanical ventilation	9.50 (8.25–10.75)	7.00 (5.00–8.00)	0.471
LOS	16.00 (15.50–16.50)	13.00 (8.50–15.50)	0.296
LOS in PICU	16.00 (15.50–16.50)	10.00 (7.00–13.00)	0.050*

*IQR* interquartile range, *PRISM* pediatric risk of mortality score, *PCIS* pediatric critical illness scoring, *Serum* BDG serum (1,3)-β-D-glucan, *LDH* lactate dehydrogenase, *CRP* C-reactive protein, *PCT* procalcitonin, *CRRT* continuous renal replacement therapy, *ARDS* acute respiratory distress syndrome, *LOS* length of stay

^*^P < 0.05

### Mixed infections and/or co-pathogens detected by mNGS

**Fig. 4:**
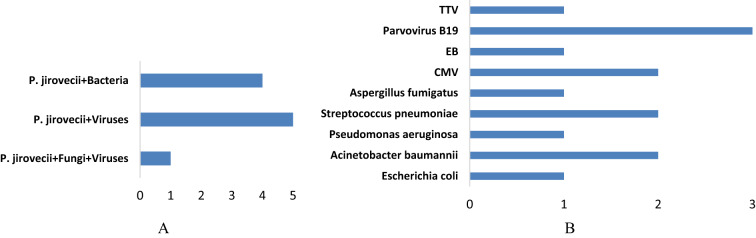
Mixed infections and co-pathogens identified by mNGS in 17 PCP patients. **A** number of PCP patients with mixed infections; (**B**) number of PCP patients infected with various co-pathogens

There are mixed infections and co-pathogens in 10 PJP patients identified by mNGS, including *Acinetobacter baumannii*, *Streptococcus pneumoniae, Aspergillus fumigatus, Pseudomonas aeruginosa, Escherichia coli, Transfusion-transmitted virus, Parvovirus B19, Epstein-barr virus* and *Cytomegalovirus* (Fig. 4). The respiratory pathogen detection results of mNGS and conventional methods are shown in Additional file 2: Table S1. The positive detection rate of various pathogens was higher using mNGS method than using conventional methods in both groups (Additional file 2: Table S2). The contents of pathogens in both groups by mNGS are shown in Additional file 2: Table S3. Only 41.2% of the observation group patients were with simple *P. jirovecii* infection while most manifested a mixed infection of *P. jirovecii* with viruses (29.4%), especially for *CMV* and *parvovirus B19* (Additional file 2: Table S4).

### Impact of mNGS on PCP patients’ antimicrobial therapy

According to the mNGS, 88.2% of the PCP critically pediatric patients modified their initial antimicrobial therapy. Trimethoprim-sulfamethoxazole was not received in 82.4% of patients until the report of mNGS results. There were 47.0% of PCP patients removing antimicrobial agents, 23.5% reducing antimicrobial spectrum and 29.4% adding antimicrobial agents (Table 6). Five cases diagnosed by mNGS were effectively treated with anti-PCP and discharged.

**Table 6:** Impact of mNGS on PCP patients’ antimicrobial therapy

Modififications(n [%])	PCP patients (n = 17) %
Remove agent	8(47.0)
Reduce antimicrobial spectrum	4(23.5)
Add agent	5(29.4)
Add TMP-SMZ	14(82.4)
Add caspofungin	1(5.9)
No change	2(11.8)

## Discussion

PCP is a life-threatening opportunistic infection and an important cause of pneumonia in immunocompromised children [31, 32]. In fact, nowadays, PCP has a fair proportion in the non-HIV immunocompromised children. The rapid detection of pathogens by mNGS is conducive to the timely diagnosis and treatment of critically pediatric patients [33–35]. In this retrospective study of 17 PCP critically pediatric patients, the dominant underlying conditions included hematologic malignancies, solid tumors, and rheumatic diseases. Similar with previous researches, leukomonocyte of PCP patients was reduced, while serum LDH and BDG were typically elevated compared to the non-PCP patients. In addition, PCP patients usually suffer from mixed infection, and the hospital mortality rate reaches 11.8%.

The mNGS technology has the advantages of unbiased sequencing by extracting total DNA or RNA (usually subsequently reverse transcribed to DNA), fragmentation, library preparation and deep sequencing from original samples. As a new pathogenic gene detection and diagnosis technology, mNGS has the advantages of rapid, comprehensive and high sensitivity in the diagnosis of PCP. For patients with impaired immune function, the probability of mixed infections of multiple pathogens in the lungs is significantly increased. Using mNGS technology to detect pathogenic microorganisms in respiratory specimens of such patients can significantly improve the sensitivity (100%), specificity (96.3%) and time efficiency of diagnosis [13]. In this study, mNGS also showed an outstanding sensitivity and specificity in diagnosing PCP, consistent with other studies [37–42].In addition, mNGS facilitates unbiased identification of mixed infections through a single experiment. Previous studies have shown that mixed infections are common in PCP [43]. Coinfections of *P. jirovecii* are considered as index of poor prognosis [44]. In our study, about 59% of PCP patients had mixed infections, and the most common mixed infections were virus and bacteria. The detection rate of total and mixed pathogens was significantly higher than that of traditional pathogen detection. Notably, the coinfection of PCP with *Parvovirus B19* is rarely reported. *Parvovirus B19* is a small non-enveloped single-stranded DNA virus of the family *Parvoviridae* [47, 48]. *Parvovirus B19* is pathogenic in human and causes a variety of clinical illnesses, including haematological diseases [49, 50]. In this study, *Parvovirus B19* was detected from a patient with T-lymphoblastic lymphoma. The other patient is with acute B lymphoblastic leukemia. Whether children with blood diseases are more likely to be infected with PCP and *Parvovirus B19* requires more cases support. Moreover, with 100% of PPV, mNGS results showed that there were 50% ARDS patients infected by *P. jirovecii*, which suggested early application of mNGS was beneficial to the rapid and proper diagnosis as well as precise treatment.

In our study, both blood and BALF samples were tested positive for *P. jirovecii* at the same time. It is widely reported that the pathogens from BALF were highly consistent with that from blood samples detected by mNGS Among that, we found the SMRNs in the BALF samples were significantly higher than that in blood samples, which suggested BALF samples were easier to detect pathogens in pneumonia. Blood samples may be a good alternative to BALF when bronchoscopic examination was infeasible.

In the present study, compared with survivors, non-survivors had higher reads of SMRNs in BALF. It is found that concurrent pathogen load correlates closely with the severity of sepsis and the survival rate of the ICU sepsis patients. It is reported that there was a positive correlation between the SMRNs of *P. jirovecii* with serum BDG both in blood and BALF samples. In other words, SMRNs may play a role in the severity of response. There was no correlation between the SMRNs of *P. jirovecii* and PRISM, PCIS, LDH as well as serum BDG in the present study. It is reported that the abundance of *P. jirovecii* in the blood of children is correlated with white blood cell counts and immune status [54]. It suggested that the SMRNs of *P. jirovecii* may be related to basal status of the patient. given that the sample size of our study was small, so that the correlation with SMRNs needs further study.Given that the sample size of our study was small, so that the correlation with SMRNs needs further study.

The unbiased broad-spectrum detection of mNGS could further provide guidance for valuable antimicrobial therapy. Based on the mNGS, 88.2% of the PCP critically pediatric patients modified their initial antimicrobial therapy and simplified the use of antibiotics. Trimethoprim-sulfamethoxazole was not received in 82.4% of patients until the report of mNGS results. Our data and previous studies showed that mNGS was excellent for the early and precise treatment of PCP. However, blind treatment based on mNGS alone is inappropriate, since mNGS technology cannot distinguish pathogens between colonization and infection. At the same time, due to its high sensitivity, false positive results may occur, while incomplete wall breaking may lead to false negative results. To our acknowledge, the occurrence and progress of infectious diseases involve the immune response of pathogens and hosts. Host response based detection has become an effective auxiliary means of traditional pathogen detection, which may improve the accuracy and efficiency of diagnosis. The combined application of host based detection and pathogen based detection is a new field worth exploring.

There were several limitations in this study. First of all, this study did not compare diagnostic performances of mNGS with PCR because PCR was not carried out routinely in our laboratory, which would be further explored in future work. Second, it was a retrospective and single-center research. In addition, the sample size of this study was small and bias was unavoidable. The diagnostic advantages of mNGS are obvious. Building a PCP diagnostic model based on mNGS and combining the characteristics of biomarkers of pathogen and host immune response will have important clinical value and potential application prospects, and will make precision treatment possible.

## Conclusion

The mNGS technology is an efficient and useful diagnostic technology for PCP in critically pediatric patients. Both blood and BALF samples for mNGS are recommended for the presumptive diagnosis of PCP. SMRNs may be relevant to the prognosis, which needs further investigation.

## Supplementary Information

**Additional file 1: Figure S1.** qPCR amplification curve and Ct value of *P. jirovecii* for mNGS validation in 5 cases.

**Additional file 2: Table S1.** The detected pthogens by mNGS and conventional clinical methods. **Table S2.** Diagnostic performance of mNGS, GMS staining,serum BDG and LDH in non-HIV-infected PJP patients. **Table S3.** The number of pathogens of different types by mNGS and conventional clinical methods. **Table S4.** The content of pathogens detected by mNGS in the observation group. **Table S5.** The qPCR and mNGS results of 17 cases in this study.

## Data Availability

The original contributions presented in the study are included in the article/Supplementary Material, further inquiries can be directed to the corresponding authors.
