# Supplementary figures and images for: Metagenomic next-generation sequencing for the diagnosis of Pneumocystis jirovecii Pneumonia in critically pediatric patients
Source: Ann Clin Microbiol Antimicrob. 2023 Jan 16;22:6. doi: 10.1186/s12941-023-00555-5 (PMC9841943; doi:10.1186/s12941-023-00555-5)

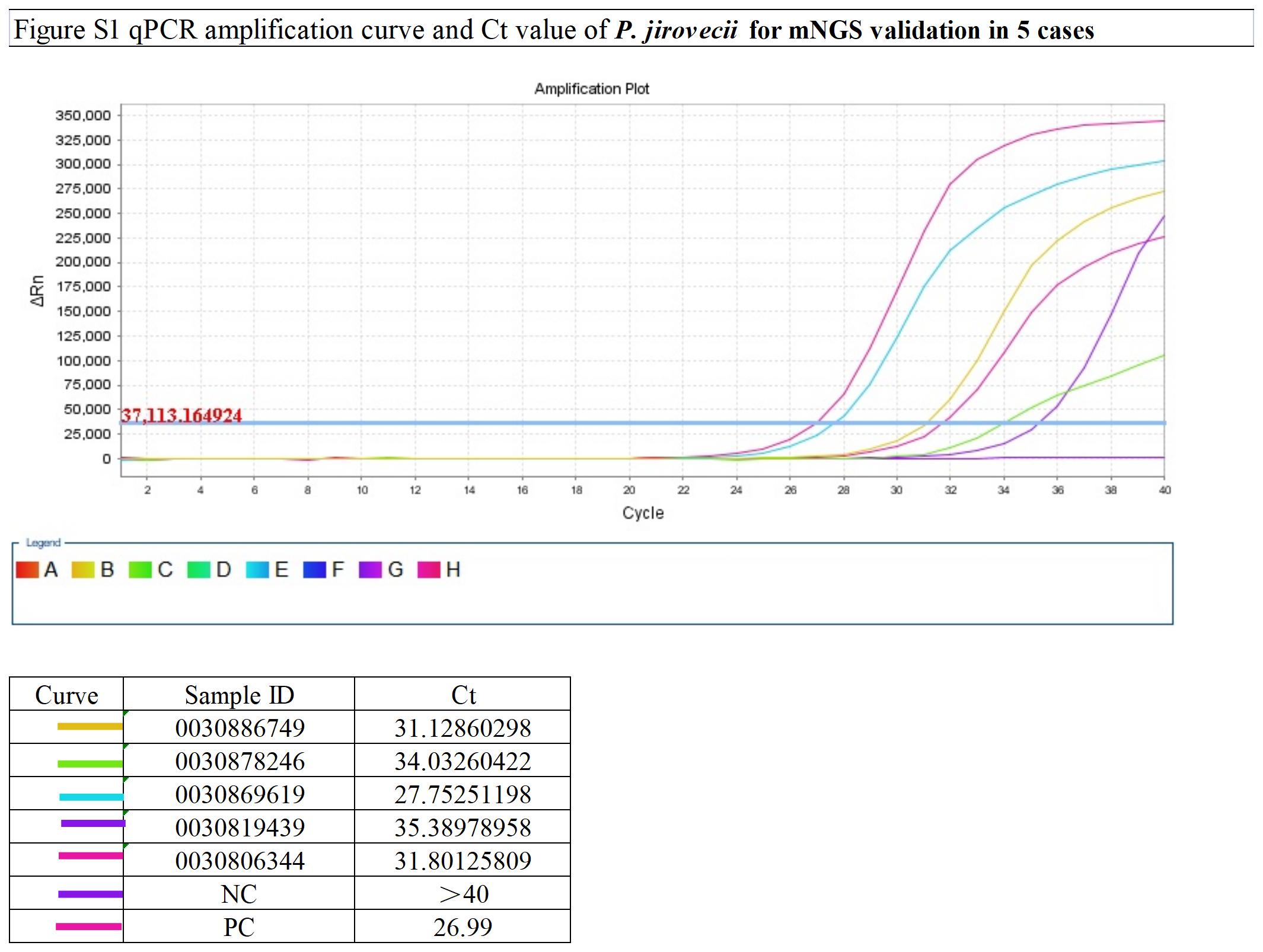

Supplement: Supplementary file 1 — Additional file 1: Figure S1. qPCR amplification curve and Ct value of P. jirovecii for mNGS validation in 5 cases. [file 12941_2023_555_MOESM1_ESM.jpg]
